# Ultra-rapid meningeal metastasis in triple-negative breast cancer: A case report

**DOI:** 10.1016/j.ijscr.2025.111574

**Published:** 2025-06-29

**Authors:** Naiqian Zhang, Shanshan Jiang, Keru MA, Lin Gu, Haoyu Chen, Zijun Zhou

**Affiliations:** a Breast Surgery Department, Jilin Cancer Hospital, Jilin, China; b The Third Affiliated Hospital of Harbin Medical University, Harbin, China

**Keywords:** Breast cancer, Triple-negative, Meningeal metastasis, Rapid progression, Case report

## Abstract

**Introduction and importance:**

Triple-negative breast cancer (TNBC) is highly invasive and poorly responsive to standard treatments, making it prone to brain metastasis, which exhibits significant intracranial invasiveness. But brain metastasis is extremely rare within one month after neoadjuvant therapy and standard modified radical surgery. Here, we report a case of a patient who, after completing neoadjuvant chemotherapy and achieving partial remission (PR), was diagnosed with brain metastasis one month post-surgery. The uniqueness of this case lies in the imaging description of a 2 cm mass, which was similarly palpable on physical examination. However, at the initial detection, skin invasion and lymph node metastasis on the ipsilateral clavicle were also identified, indicating highly aggressive behavior. For such highly invasive cases, intensified neoadjuvant therapy and close monitoring should be considered.

**Case presentation:**

We report a female patient who despite achieving a partial response (PR) after neoadjuvant chemotherapy was found to have brain metastatic lesions due to obvious symptoms of meningeal metastasis only one month after surgery. The patient's meningeal metastasis remained stable only after undergoing whole-brain radiotherapy and intrathecal injection of chemotherapy drugs.

**Clinical discussion:**

Supraclavicular lymph node metastasis is a high - risk factor for metastasis in triple - negative breast cancer. Effective neoadjuvant therapy is a factor contributing to a favorable prognosis. This patient exhibits both of these characteristics. For this particular patient, a head MRI may enable earlier detection of brain metastases.

**Conclusion:**

For high - risk triple - negative breast cancer patients, the risk of brain metastasis should be closely monitored.

## Highlights

• A patient with highly invasive triple-negative breast cancer (TNBC) and small primary tumor presented with supraclavicular lymph node metastasis.
• Neoadjuvant therapy induced partial response (PR).
• Postoperative rapid progression to symptomatic leptomeningeal metastasis occurred.

## 1 Introduction

Triple-negative breast cancer (TNBC) has a higher risk of recurrence and metastasis compared to other molecular subtypes [1]. A comprehensive meta-analysis of large-scale studies on TNBC indicates that the median time from the initial diagnosis of early disease to the development of metastasis is 18.7 months (range: 1.4–97.7 months). In patients who developed brain metastasis (BMs), the median time from initial diagnosis to the appearance of the first metastatic site was 20 months (range: 2–68 months), and the median time to brain metastasis was 27 months (range: 6–93 months) [2]. Therefore, the occurrence of super-rapid meningeal metastasis during the adjuvant therapy phase of breast cancer is extremely rare. Moreover, the characteristics of super-rapid meningeal metastasis in TNBC are rarely reported, highlighting the urgent need to further investigate the biological features of such high-risk tumors. This article presents a case of a patient who developed brain metastasis after surgery, before the planned adjuvant radiotherapy. This is the first reported case of TNBC metastasis occurring despite effective treatment and prior to completion.

## 2 Case report

**Fig. 1:**
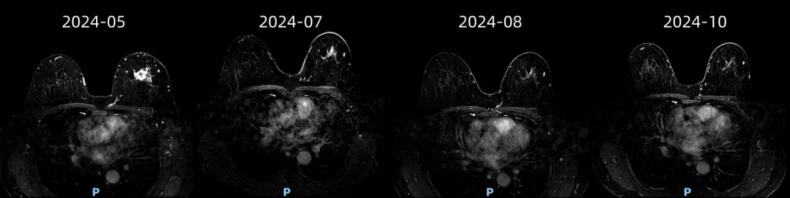
After the start of neoadjuvant therapy, breast MRI should be rechecked every two cycles, and the curative effect is partially relieved.

A 54-year-old female patient discovered a painless lump in her left breast incidentally. She did not initially pay much attention to it and sought medical attention two months after the onset of symptoms, on May 28, 2024, at the second hospital of Jilin University The patient underwent a biopsy of the left breast and left axillary lymph nodes. After the biopsy, the pathological examination confirmed invasive ductal carcinoma grade 2. Immunohistochemistry (IHC) results were as follows: estrogen receptor (ER)-, progesterone receptor (PR)-, human epidermal growth factor receptor 2 (HER-2)-, Ki67 80 %, P63-, CK14 partially positive, CK5/6-. Cancer was found in the left axillary puncture. Later, she visited our hospital and a puncture of the left supraclavicular area indicated the presence of cancer cells. The patient has no family history of breast cancer or ovarian cancer, no bad habits such as smoking or drinking, and works as a manual laborer. The patient had local skin edema accompanied by partial orange peel-like changes. A systematic whole-body examination showed no distant metastasis. The clinical stage was cT4bN3cM0, stage IIIc. The patient was diagnosed at an advanced clinical stage. After a multidisciplinary consultation, it was recommended to initiate neoadjuvant therapy. Due to financial reasons, the patient was unable to receive immunotherapy and instead underwent 6 cycles of neoadjuvant chemotherapy with the TA regimen (albumin-bound paclitaxel + pirarubicin). The clinical response was partial remission (Fig. 1). During the neoadjuvant treatment, the patient demonstrated good compliance, experiencing only grade 3 myelosuppression and mild peripheral neuropathy, which were relieved after symptomatic treatment.

Before the operation, the patient underwent a systematic whole-body examination. The left supraclavicular lymph nodes showed no obvious enlargement and no distant metastasis. The preoperative clinical stage was cT2N2M0, stage IIIa. On October 23, 2024, a modified radical mastectomy for breast cancer was performed under general anesthesia. Postoperative pathology: (After neoadjuvant treatment of the left breast gland) Invasive carcinoma, unable to be graded. The density of residual tumor cells was 5 %. The tumor range was about 7 mm, presenting as small clusters or single cells scattered in the fibrous stroma. Tumor parenchymal changes such as eosinophilic change of tumor cells, cell vacuolation, nuclear enlargement/atypical nuclei were observed. No definite vascular tumor embolus, no definite nerve invasion, no epidermal invasion were found. The nipple, circumferential margin, skin margin, and basal margin were free of cancer. Lymph nodes: 8/13 in group I, 2/4 in group II, 0/2 in group III, with cancer metastasis. The maximum diameter of the metastatic foci was 7 mm, and 10 of them showed a chemotherapy response. The stage was ypT1bN3aMx. Immunohistochemistry results: ER-, PR-, HER-2 (0/negative), Ki67 (+), CK(+), P63(−). (Note: There were very few residual tumor cells, and the immunohistochemistry results were for reference only.)

**Fig. 2:**
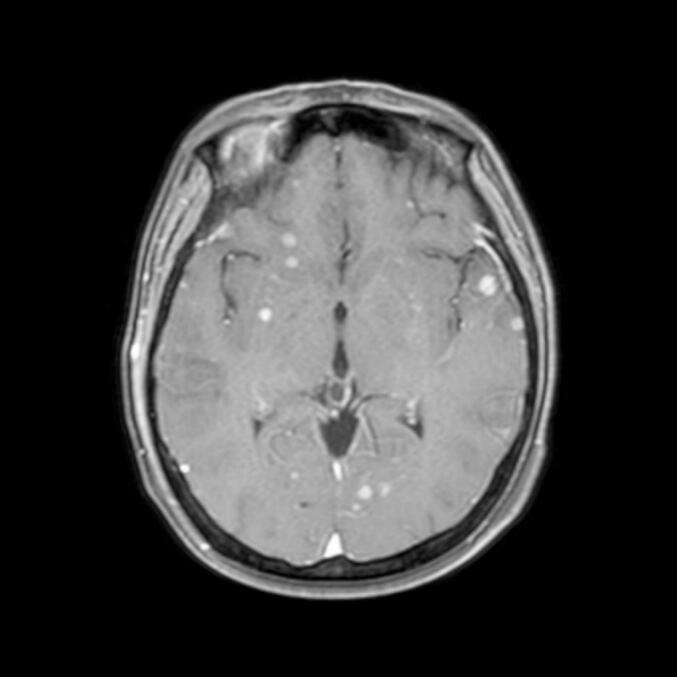
In the left temporal lobe had a long diameter of about 0.7 cm, suggesting multiple intra-cerebral and meningeal metastases.

The patient recovered well after the operation and was discharged on October 31, 2024, without obvious discomfort symptoms, waiting for postoperative radiotherapy. On November 23, 2024, due to nausea, dizziness, and headache, the patient underwent a plain magnetic resonance imaging (MRI) scan of the head + diffusion-weighted imaging which suggested multiple abnormal signal shadows in the brain, considered as multiple metastatic tumors. On November 25, 2024, the patient visited our hospital for a follow-up. An enhanced MRI scan of the head suggested nodular, linear, and patchy abnormal enhancement shadows in the brain. The largest nodular enhancement in the left temporal lobe had a long diameter of about 0.7 cm, suggesting multiple intra-cerebral and meningeal metastases (Fig. 2). The cerebrospinal fluid exfoliated cell examination was completed, and a diagnosis of meningeal metastasis was confirmed. Palliative radiotherapy for the head was given. The radiotherapy modality used was volumetric modulated arc therapy with image-guided radiation therapy (VMAT-IGRT).

**Figs. 3, 4:**
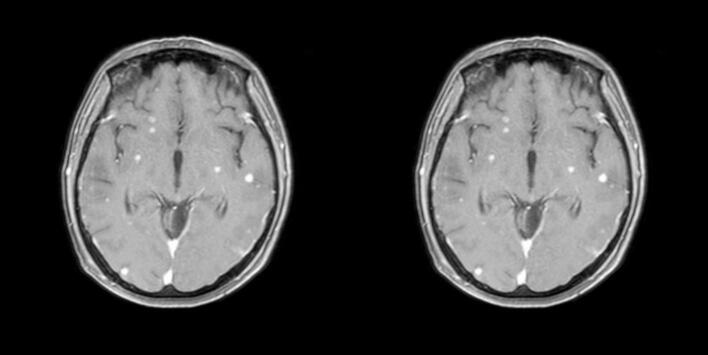
The metastatic tumors had shrunk compared with before.

The total radiotherapy dose was 42 Gy/15 fractions/21 days (for multiple brain and meningeal metastatic tumors) and 37.5 Gy/15 fractions/21 days (for the whole brain). Meanwhile, 3 cycles of intrathecal methotrexate chemotherapy were given. During the radiotherapy, treatments for reducing intracranial pressure and relieving vomiting were carried out. After the treatment, the patient's nausea, dizziness, and headache were significantly relieved. A reexamination of the head MRI suggested that the metastatic tumors had shrunk compared with before (Figs. 3, 4). The patient was instructed to continue intrathecal chemotherapy regularly and undergo close follow-up examinations.

This case has been reported in line with the SCARE criteria [3].

## 3 Discussion

The results and reports of multiple large-scale international studies on TNBC cohorts indicate that the median time interval between breast cancer diagnosis and the occurrence of distant metastasis is from 19.7 months to 31.2 months [4,5]. Brain metastasis, the most severe form of distant metastasis, significantly shortens the event-free survival (EFS) and overall survival (OS) of breast cancer patients. Currently, there are still no effective treatment options for advanced triple-negative breast cancer. Therefore, early detection of brain metastases and timely intervention are crucial.

Previous studies have suggested that patients with poorer pathological types, vascular invasion, higher tumor grades, increased Ki67 expression levels, and later T and N stages are more likely to have postoperative recurrence and metastasis, resulting in a poor prognosis [6]. For TNBC patients undergoing neoadjuvant chemotherapy, only the N3 stage is a risk factor for rapid recurrence [7].

In neoadjuvant treatment, the mainstream view is that the efficacy of neoadjuvant chemotherapy is the best biomarker for TNBC [[8], [9], [10]]. Therefore, effective neoadjuvant chemotherapy is a protective factor for TNBC patients against rapid recurrence. Patients with poor neoadjuvant treatment efficacy are more likely to have rapid recurrence. The rapid recurrence risk of patients with effective neoadjuvant chemotherapy is 0.059 times that of patients with ineffective neoadjuvant chemotherapy [11]. However, in this case, brain metastasis occurred despite the effectiveness of neoadjuvant therapy, with an exceptionally rapid progression. What makes this case unique is that the imaging revealed a relatively small tumor, yet it invaded the local skin and involved lymph node metastasis in the supraclavicular region, demonstrating a high metastatic potential.

In this case, we used plain computed tomography (CT) scans of the brain to evaluate the condition during both the neoadjuvant and preoperative assessments. Head CT has good cost-effectiveness and is convenient and fast for examination. In the Chinese version of the Guidelines for the Diagnosis and Treatment of Breast Cancer by the Chinese Breast Cancer Society (CBCS), head MRI and CT are given the same recommendation level in neoadjuvant screening. MRI can provide more detailed details, localization, and characterization of brain metastases, mainly due to its high soft tissue contrast and a large number of MRI sequences available for characterizing intracranial lesions [2]. However, in clinical practice, there is still controversy over which imaging technique should be used for screening brain metastases. In patients who are strongly suspected of having a known malignant tumor in clinical practice and in those where CT cannot determine whether there is metastasis, using magnetic resonance imaging (MRI) to detect lesions is a suitable strategy.

## 4 Conclusion

TNBC still carries the possibility of super-rapid brain metastasis even after neoadjuvant therapy, especially in cases with smaller tumors that exhibit significant invasiveness or metastatic characteristics. It is recommended to strengthen cranial MRI monitoring to facilitate early detection of brain metastases and timely intervention, thereby improving patient prognosis.

## Consent

Written informed consent for publication of their clinical details and/or clinical images was obtained from the patient.

## Ethical approval

Science and Technology Ethics Committee of Jilin Cancer Hospital，Jilin, CHINA on 17 January 2025. Approval Number: 202501–004 - 01 (Thesis),

## Funding

None.

## Author contribution

Naiqian Zhang writing-original draft

Shanshan Jiang writing-original draft

Zijun Zhou writing-review editing

Keru MA writing-review editing

Lin Gu writing-review editing

Haoyu Chen writing-review editing.

## Guarantor

Naiqian Zhang.

## Research registration number

None.

## Conflict of interest statement

The authors declare having no conflicts of interest for this article.
